# Aging in Biometrics: An Experimental Analysis on On-Line Signature

**DOI:** 10.1371/journal.pone.0069897

**Published:** 2013-07-23

**Authors:** Javier Galbally, Marcos Martinez-Diaz, Julian Fierrez

**Affiliations:** Biometric Recognition Group-ATVS, Universidad Autonoma de Madrid, Madrid, Spain; University of Adelaide, Australia

## Abstract

The first consistent and reproducible evaluation of the effect of aging on dynamic signature is reported. Experiments are carried out on a database generated from two previous datasets which were acquired, under very similar conditions, in 6 sessions distributed in a 15-month time span. Three different systems, representing the current most popular approaches in signature recognition, are used in the experiments, proving the degradation suffered by this trait with the passing of time. Several template update strategies are also studied as possible measures to reduce the impact of aging on the system’s performance. Different results regarding the way in which signatures tend to change with time, and their most and least stable features, are also given.

## Introduction

Due to the fact that biometrics, as an automatic means of human recognition, constitutes a relatively novel field of research [1], up to date most efforts undertaken by the different parties involved in the development of this technology (researchers, industry, evaluators, etc.) have been mainly focused on the improvement of its performance (i.e., finding novel methods to obtain lower error rates) [2], [3]. As a consequence, other important aspects closely related to this type of systems such as the performance degradation effect known as *aging* have been left partially uncovered [4].

Although there always exists a certain variability among biometric samples of one given user (even when they have been acquired successively) [5]–[7], in biometrics the term *aging* is generally used to refer to the gradual decrease in a system performance caused by the changes suffered by the users' trait in the long-term (which cannot be avoided as is inherent to human nature) [8]. These changes provoked by age, entail that, after a sufficiently long period of time, the initial enrolment template of a certain subject substantially differs from his current biometric samples, producing this way lower similarity scores and increasing the error rates of the system. Thus, aging may be considered as a especial type of large intra-user variability (i.e., variability within the samples of the same user) caused by the inherent transformations of the human body or behavior over time.

The amount of time required for the stored template to become obsolete varies for each biometric trait depending, among other external aspects, on its own degree of permanence [4]. Thus, biometric traits such as the fingerprints or the iris are more stable over time, while others, such as the face (especially at early stages of life) or the signature, are much more sensitive to relatively short time variations. Furthermore, not every biometric system will be affected in the same way by aging, as the features extracted from one given biometric trait are not necessarily all equally robust to the passing of time. In this context, the effect of aging should be analyzed in a wide range of recognition systems working on a certain individual biometric modality in order to determine the approximate period of time in which the performance of that given trait will be consistent, before its discriminant capabilities start to drop.

In addition, once the consistent-performance time interval for a given recognition system has been set, an analysis of the best approach to overcome the effect of aging should also be carried out. Among the different palliative methods that have been proposed in the literature, the ones that have received more attention from researchers and industry are the automatic template update strategies [9], [10]. These schemes use some type of target function (e.g., quality measure, similarity score) to automatically select from the most recent biometric samples given by the user to access the system, those which are most suited to be used to recompute (update) the subject's enrolment template.

In this context, for the definitive introduction of the biometric technology in the security market, it is of utmost importance to take into account the problem of aging in practical biometric applications, and to implement strategies that compensate the gradual drift of their performance so that their valid life period (in which they are competitive) is increased.

However, in spite of their importance, studies regarding aging and template update are difficult to be carried out due to the lack of long-term biometric data. It is not easy to find databases where a statistically significant group of people have been captured over a sufficiently long period of time [11]. Furthermore, the acquisition process of such a database should be carried out under almost identical conditions (in terms of acquisition devices, level of control, supervision, etc.) so that the differences in the system performance can be attributed to the elapse of time and not to the variability produced by other external factors.

It has not been until recently that different European and national efforts have led to the acquisition of compatible (regarding certain traits) multimodal databases with a relatively large number of common users which have been captured in different sessions over a several year time span. Some examples include the Biosec [12], BiosecurID [13] and Biosecure [14] projects. For the current work, the signature modality of this common subset of users has been used to generate a new Long-Term dynamic signature dataset which has been deployed to analyze the effect of aging on three competitive on-line signature verification systems working on totally different features and matchers. In addition to the study of the signature performance stability over time, several template update strategies have also been explored in order to assess their efficiency as a way to maintain the consistency of the system performance in the long-term. Furthermore, several experiments regarding the changes suffered by signatures with time and their most/least robust features have also been carried out.

This way, although some novelty may be found in the algorithms and techniques used in the experiments, the most relevant contributions of the present work lie on: 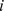) the comprehensive revision of the state of the art in aging related problems; 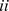) the presentation of the first dataset where the signature of different subjects may be tracked over more than a year; 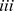) the rigorous methodology followed to reach the experimental results, which may be generalized in the future for similar aging studies focused on other biometric traits; 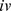) the experimental findings and practical conclusions extracted from them, which help to shed some light into the difficult problem of handwriting evolution over time.

The rest of the article is structured as follows. After the introduction, a selection of the most important related works may be found in Sect. The on-line signature Long-Term DB used in the experiments is presented in Sect. The experimental protocol followed is described in Sect. while results are given in Sect. Conclusions are finally summarized in Sect.

## Related Works

In the literature there exist different works where the aging of human biometric traits has been studied from a medical point of view [15]–[18], to help in the early diagnosis of diseases [19], or even its forensic implications [20], [21]. However, not many studies can be found where aging is analyzed from a pure biometrical perspective (two surveys of these works were recently published in [8] and [22]). Furthermore, almost all of these aging biometric works are related to the face modality, but, to the best of our knowledge, none of them have been focused on the study of the signature trait.

Among these face related contributions, there are works dealing with different aspects of aging, for instance, its effect on the performance of face verification systems [23], [24], methodologies for the synthetic simulation of age [25], [26], approaches for the compensation and modeling of the aging effect [27], automatic age estimation methods [28]–[30], or descriptions of long-term facial databases [11]. All this interest in the study of the effect of time on face recognition, led in 2004 to the creation of a research group specialized in the analysis of the different factors related to face aging [31].

Outside the face trait, Modi *et al.* studied the correlation between the quality of fingerprint samples and the age of the users that produced them, and its impact on the final performance of fingerprint recognition systems [32], [33]. In the same direction as the fingerprint works by Modi *et al.*, several studies have analyzed the degree of the signing/drawing skill of people belonging to different age groups, their ability to repeat certain valuable recognition features and their vulnerability to eventual imitators [34]–[37]. Although all these works study an interesting issue related to aging, they are not equivalent to the analysis carried out in the present work, as they do not track individuals over a significant period of their life, but they are focused on establishing a relationship between a certain group of people (e.g., the elderly, youngsters) and a given characteristic (fingerprint quality or signing skill) of their biometric samples (e.g., the elderly-bad quality-poor skill, youngsters-good quality-high skill).

In addition to the aforementioned works, several authors have also addressed aging-related problems (such as age estimation or age modeling), generally using relatively short-term data, in biometric traits such as the handwriting [38], the voice [39], [40], or even the gait [41].

Although it cannot be strictly considered as aging, several works have analyzed the short term variability of signatures using samples captured in the same session (intra-session variability, within minutes), or in different sessions (inter-session variability, within days/weeks) of a regular acquisition campaign [35], [42]. In these cases, the differences in the systems performance can be attributed more to the inherent variability of the biometric samples (inter and intra-user short term variability) than to a real process of aging, as the time interval between samples is in general too short [5], [43].

Regarding strategies that try to minimize the effect of aging, among other possibilities such as using age invariant features [35], or compensating age changes [44], most efforts have been focused on the study of template update techniques [10](i.e., using the most representative recent test samples of a user to update his enrolment template). In this field, different fully unsupervised or semiautomatic approaches have been proposed for the fingerprint trait [45], [46], for face-based systems [47], or even in multimodal biometric applications [48].

## The On-Line Signature Long-Term Database

The dataset used in the experimental section of this work comprises the on-line signature data of the 29 common users to the BiosecurID and the Biosecure databases. These two signature subsets, which were acquired in a 15 month time span, present some unique features that make them especially suited for the aging evaluation performed in the present work.

**Figure 1:**
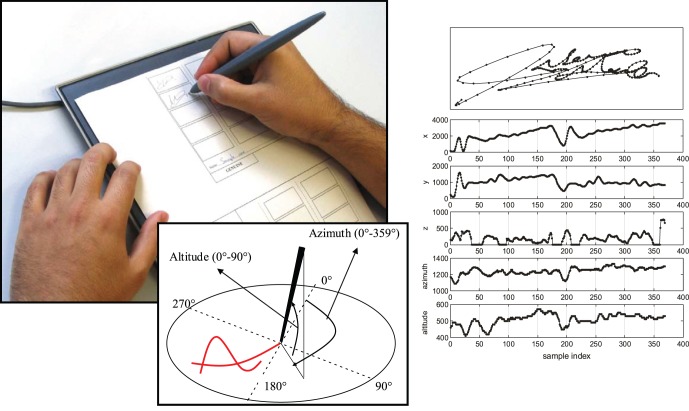
Example of a signature acquisition for the Signature Long-Term DB using the Wacom Intuos 3 digitizing tablet and a paper template with a delimited signing area for each sample.

**Figure 2:**
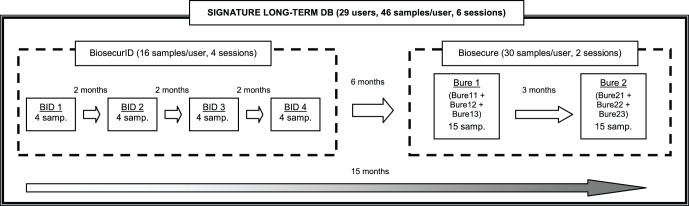
General time diagram of the different acquisition sessions that conform the Signature Long-Term Database.

- **The BiosecurID Signature Subset** [13]. It comprises 16 original signatures and 12 skilled forgeries per user, captured in 4 separate acquisition sessions (named here BID1, BID2, BID3 and BID4). The sessions were captured leaving a two month interval between them, in a controlled and supervised office-like scenario. Users were asked to sign on a piece of paper, inside a grid that marked the valid signing space, using an inking pen. The paper was placed on the Wacom Intuos 3 pen tablet that captured the time signals of each signature at a 100 Hz sampling rate (trajectory functions 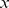 and 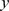 with an accuracy of 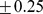
mm, and pressure function 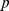 with a precision of 1024 pressure levels). All the dynamic information is stored in separate text files following the format used in the first Signature Verification Competition, SVC [49]. All the acquisition process was supervised by a human operator whose task was to ensure that the collection protocol was strictly followed and that the captured samples were of sufficient quality (e.g., no part of the signature outside the designated space), otherwise, the donor was asked to repeat a given signature. In a second stage, the database was validated by a signature expert to avoid unwanted mistakes. For further details on the acquisition and validation process we refer the reader to [13]. See Fig. 1 for an acquisition example.
- **The Biosecure Signature Subset** [14]. This dataset was captured 6 months after the BiosecurID acquisition campaign had finished (the time sequence of the two databases is shown in Fig. 2). It comprises 30 original signatures per user, and 20 skilled forgeries, distributed in two acquisition sessions separated three months (named here Bure1 and Bure2). The 15 original samples corresponding to each session were captured in three groups of 5 consecutive signatures with an interval of around 15 minutes between groups (named here Bure11-12-13 and Bure21-22-23, respectively). The signature dataset was designed to be fully compatible with the BiosecurID one. The acquisition scenario and protocol are almost identical: as in the BiosecurID case, users had to sign using an inking pen on a piece of paper with a restricted space, placed over the Wacom Intuos 3 pen tablet. The dynamic information stored is the same as in BiosecurID and following also the SVC format. The supervision and validation of the database was very similar as well to that followed in BiosecurID, with a human operator controlling the acquisition process and an expert doing a posterior verification of the data (a complete description of both tasks is given in [14]).

For the final dataset used in the present work, only the original signatures were considered. This way, it comprises 1,334 signatures coming from the 29 common users of the two databases with 46 samples per user (16 from BiosecurID, and the remaining 30 from Biosecure) which are distributed in 6 sessions (BID1-2-3-4 and Bure1-2) according to the general time diagram shown in Fig. 2.

It constitutes the first signature dataset where we can track, over a 15 month time span (as there are 6 almost uniformly distributed acquisition sessions in this interval), the signature of a given user, and assess if that period of time is sufficient to detect a decrease in the verification performance of signature-based biometric systems. Furthermore, as all the samples of the same subject have been acquired under almost identical conditions we may discard external factors as the cause of a possible degradation in the recognition rates.

All users in the database are Spanish, white Caucasian with higher level education, between 20 and 51 years of age. In particular, the age distribution of the subjects is: 24 donors between 18 and 25; 3 donors between 25 and 45; and 2 donors above 45 years old. The gender distribution within the database is quite balanced with 11 women and 18 men.

It should also be noted that all the users included in the database may be considered as adults in terms of writing. This means that their signature is a well learned sequence of movements which may be considered as permanent and that has already gone through the transitional learning period which usually happens in the youth. The effect of aging during the time in which the signature has not yet been fully fixed should be much greater and would be the subject of future work.

**Figure 3:**
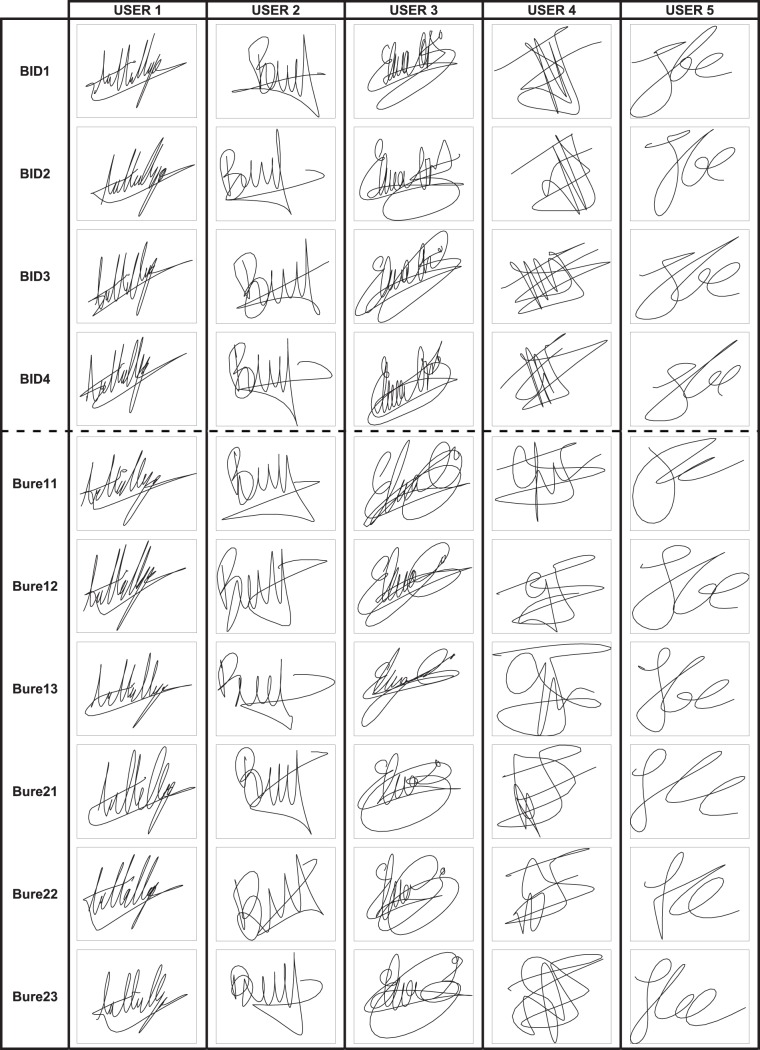
Typical samples that can be found in the Signature Long-Term DB. Each signature corresponds to each of the acquisition sessions of five different users.

Some typical examples of the signatures that can be found in the different sessions comprised in the Signature Long-Term DB are shown in Fig. 3.

The Signature Long-Term DB is publicly available for research purposes at the Biometric Recognition Group-ATVS website [50].

## Experimental Protocol

The experimental framework has been designed to evaluate the effect of aging on the performance of signature-based systems and to assess the stability of signatures through time. In particular, five different objectives are pursued in the experiments, which may be divided into two main groups:

- **Signature recognition performance.** On the one hand, 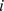) to evaluate the loss of performance of different competitive signature recognition systems as a consequence of the changes suffered by the signature trait with time (i.e., aging); 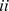) to determine the dependencies of this performance degradation (e.g., signature-dependent *vs* user-dependent); and 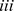) to assess the efficiency of different template update approaches to thwart this effect.
- **Signature evolution.** On the other hand, 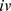) to determine which are the changes that entail the previously evaluated decrease in the signature recognition performance; and 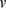) to establish which are the most stable features in the signature trait.

In order to achieve these goals the experimental protocol includes two groups of tests which are described in the next sections.

### Tests 1: Signature Recognition Performance

The first objective of this group of experiments is to evaluate the degree of aging that may be observed in the recognition performance of signature-based systems. The results will also shed some light on the user- and signature-dependency of aging, that is, if certain type of signatures are more prone to worsen their performance in the long term, or if this only depends on the signer (second objective).

The third objective of these tests is to analyze different template update approaches that can help to reduce the performance deterioration that signature recognition systems suffer with time.

In order to reach these goals, several sets of genuine matching scores (i.e., those computed between samples of the same user and therefore affected by aging) are computed on the Signature Long-Term DB simulating two different scenarios:

- **Aging experiments: Fixed template and varying test.** In this case the user models enrolled to the system are always computed using the same samples (i.e., those belonging to the first session of the Signature Long-Term DB, BID1), while the test signatures are taken from the following sessions (BID2-3-4 and Bure1-2).
- **Template update experiments: Varying template and fixed test.** In this case the test samples are always taken from session Bure13, while the enrolled models are updated with signatures coming from different previous sessions (BID2-3-4 and Bure11-12).

**Figure 4:**
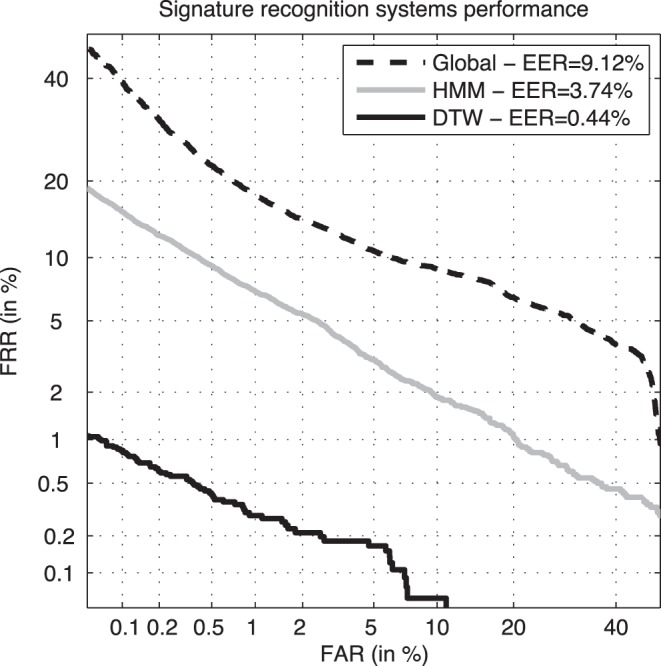
Performance of the three signature recognition systems used in the experiments, considering 4 signatures for enrollment, and evaluated on the BiosecurID DB.

As mentioned in Sect. 0, not all the systems working on a given trait are necessarily affected in the same way by aging. In order to account for possible differences, we have carried out this set of experiments on three different competitive on-line signature verification systems using totally diverse feature sets (feature- and function-based) and matchers (Mahalanobis distance, Hidden Markov Models, and Dynamic Time Warping). A brief description of each of the three systems is given next, while their DET curves evaluated on the BiosecurID DB (as an indication of their recognition capabilities) are shown in Fig. 4:

- **System A: function-based+HMM.** This function-based verification system applies a regional approach using a statistical model built using Hidden Markov Models (HMMs) [51] to a set of 10 time sequences selected applying the Sequential Forward Floating Selection (SFFS) algorithm [52] to the total set of 34 functions defined in [53]. This subset of 10 signals are derived from the coordinate (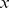 and 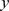) and pressure (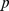) functions, while no pen inclination signals are used as its utility for automatic signature recognition is at least unclear [54]. After some preprocessing (position and rotation alignment) and the computation of the 10 functions, similarities are computed using 12 left-to-right HMM states and mixtures of 4 Gaussians per state. This system participated in the Signature Verification Competition 2004 with very good results [49], and the general configuration is detailed in [54].
- **System B: feature-based+Mahalanobis distance.** This system models the signature as a holistic multidimensional vector composed of the best performing 40-feature subset extracted in [55] from the total set of 100 global features described in [56] which may also be found for quick reference in Appendix S1 (submitted as a supporting information file of the present article). In the present study, we used this 40-feature representation of the signatures normalizing each of them to the range [0,1] using the tanh-estimators described in [57]. Finally, the similarity scores are computed using the Mahalanobis distance between the input vector and a statistical model of the client estimated using a fixed number of training signatures.
- **System C: function-based+DTW.** In this function-based local approach a subset of 9 time functions (selected using SFFS from the total 34 feature set as in the case of system B) are directly matched using the elastic technique known as Dynamic Time Warping (DTW) [58]. Dynamic Time Warping is an application of Dynamic Programming to the problem of matching time sequences of different lengths, thus, the goal of DTW is to find an elastic match among samples of a pair of sequences that minimize a given distance measure. In this particular implementation, which is described in [59], we use the Euclidean distance as the measure to be optimized and only three correspondences among samples of the compared sequences are allowed, using symmetrical weighting factors. Although the DTW algorithm has been replaced by more powerful ones such as HMMs or SVMs for speech applications, it remains a highly effective tool for signature verification as it is best suited for small amounts of training data, which is a common case in signature verification. As an example, the DTW implementation used here was the winner of the BioSecure Signature Evaluation Campaign 2009, outperforming other systems based on HMMs and global features [60].

### Tests 2: Signature Evolution

In this case, the aim of the experiments is to give some indication on whether there is a common trend in the evolution through time of signatures coming from different users (objective four), and if there are certain type of features (e.g., static *vs* dynamic) which are more stable (objective five).

To reach these objectives, the Signature Long-Term DB is parameterized using the set of features described in [56]. In that work, a set of 100 global features (i.e., features computed over the entire signature as opposed to a localized area of interest) was proposed as a compact representation of the information comprised within a signature (see Appendix S1). This 100-feature set may be divided into two classes according to the information contained by each of the parameters, namely: static or dynamic. All the features assigned to each class are specified in Table 1 (the numbering criterion is the same used in [56]).

**Table 1:** Division of the feature set introduced in [56] (given also in Appendix S1) according to the type of information they contain.

	Features #
Static	2,7,8,12,15–19,24,27–28,30,34–37,43,46,51,53–57,61,63,65–67,
	70–73,75,77–78,84,86,93,95,97–99.
Dynamic	1,3–6,9–11,13–14,20–23,25–26,29,31–33,38–42,44–45,47–48,50,52,58–60,
	62,64,68–69,74,76,79–83,85,87–92,94,96,100.

## Results

The results obtained for the two sets of experiments described in Sect. are presented in the next sections.

### Tests 1: Signature Recognition Performance

As mentioned in Sect. aging may be defined as the loss of performance experimented by biometric systems due to the transformations suffered by biometric traits in the long term. With this in mind, the questions raised in this section are: Is aging present in the signature trait? To what extent? Are some users more prone to be affected by aging than others? How can it be corrected?

In order to give an answer to these questions, several sets of genuine scores (i.e., those affected by aging) are computed in order to evaluate the performance of signature recognition systems.

Before presenting the results, it is very important to notice that, given a fixed set of impostor scores, the best possible performance results are reached when the genuine similarity score distributions have a mean value as high as possible and a variance as low as possible. Therefore, a worsening of the systems performance with time (i.e., aging) may be caused by two factors: 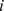) a decrease of the genuine distributions mean value, or 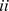) an increase of the genuine distributions variance.

#### Objective 1: Aging analysis

As mentioned before, these experiments are aimed at estimating the impact of aging on signature recognition systems. For this purpose, the enrolled models of the 29 users present in the Signature Long-Term DB are trained using the 4 signatures corresponding to the first session (BID1). Then, the sets of genuine and impostor scores are computed as follows:

- Genuine scores are generated matching the models against the signatures of the following 5 sessions: BID2-3-4 and Bure1-2. This way, for each user 5 different sets of genuine scores are computed: BID1 vs BID2, BID1 vs BID3, BID1 vs BID4, BID1 vs Bure1, and BID1 vs Bure2 (see Table 2).
- On the other hand, the same set of impostor scores is used for all the experiments A–E (i.e., we assume impostor signatures may come from any of the acquisition sessions as they are not affected by aging). To compute the set of impostor scores one signature from each session of the rest of the users is matched against the enrolled model of the subject at hand, leading this way to a total 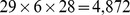 impostor scores.

**Table 2:** Enrollment and test signatures used to compute the genuine scores in the aging experiments.

	Aging Experiments	
	Enrollment	Test
Exp. A	BID1 (4 sig.)	BID2 (4 sig.)
Exp. B	BID1 (4 sig.)	BID3 (4 sig.)
Exp. C	BID1 (4 sig.)	BID4 (4 sig.)
Exp. D	BID1 (4 sig.)	Bure1 (15 sig.)
Exp. E	BID1 (4 sig.)	Bure2 (15 sig.)

As the impostor score distribution is fixed for all the scenarios, any changes observed in the performance of signature recognition systems among experiments A–E will be caused by changes in the genuine score distributions.

**Figure 5:**
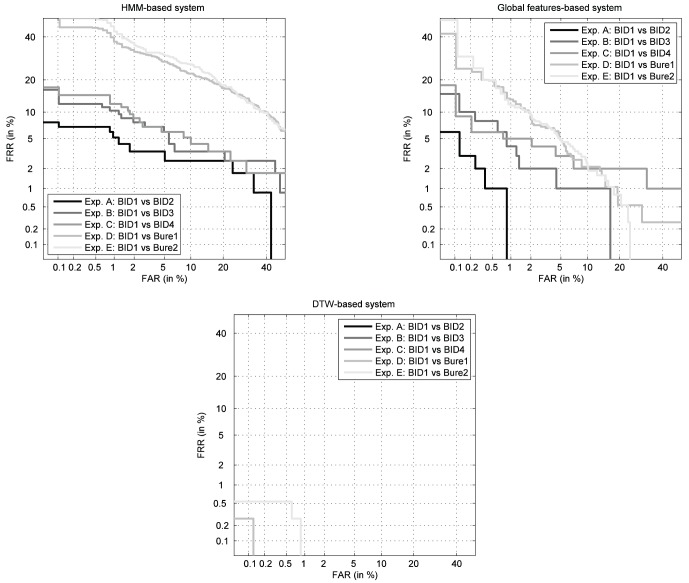
Performance evolution of the three signature recognition systems considered in the experiments. For the DTW-based system only two curves appear as for experiments A–C its EER is close to zero. The EER for the three systems and for the different experiments are reported in Table 3.

The DET (Detection Error Trade-off) curves obtained with the aforementioned genuine and impostor scores for the five scenarios (A–E) and for the three recognition systems are shown in Fig. 5. A darker gray level corresponds to a better performance of the evaluated system. It may be observed that, as the test signatures are more distant in time from those samples used for enrollment, the performance of all the three systems drops. For completion, the Equal Error Rate (EER) corresponding to the curves shown in Fig. 5 is given in Table 3.

**Table 3:** EER for the aging experiments defined in Table 2. The whole DET curves for these experiments are shown in Fig. 5.

	Aging Experiments - EER (%)				
	Exp. A	Exp. B	Exp. C	Exp. D	Exp. E
HMM-based	3.21	5.52	5.63	22.67	27.83
GF-based	0.96	2.01	4.16	4.93	4.96
DTW-based	0.0	0.0	0.01	0.12	0.51

**Figure 6:**
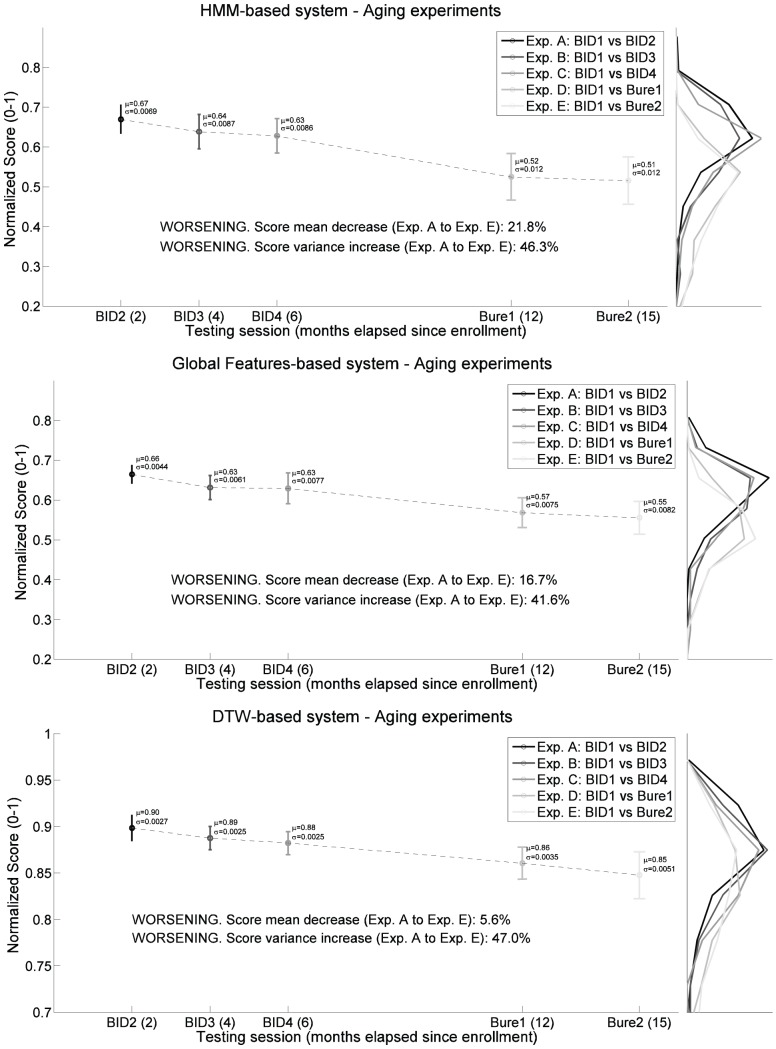
Evolution through time of the mean (circles) and variance (vertical lines) of the genuine score distributions (in vertical on the right) for the three systems considered in experiments A–E. A darker gray level represents a better performance of the given system.

In order to further analyze this performance loss, in Fig. 6 we show the evolution of the genuine scores when the test signatures move away (in terms of time) from the model. The distributions for each of the five sets of genuine scores are depicted on the right planes (in vertical) with a darker gray representing a better performance. On the left planes we can see the mean (circles) and variance (vertical lines) for each of the five distributions. Several observations can be extracted from the results shown in Figs. 5 and 6:

- The performance of the three systems consistently decreases as the testing signatures move away from the model (the DET curves in Fig. 5 are further away from the origin), which means that the users discriminant power decreases with time or, in other words, that all the three recognition approaches are affected by aging. The previous observation indicates that this effect is not particular of a certain signature recognition technology, but that, as expected, it is inherent to the signature trait itself.
- Not all the systems are affected in the same way by the passing of time, that is, not all the curves in Fig. 6 present the same decreasing slope. In particular, the system based on DTW presents a decrease in the average genuine score between the first and the last test set of signatures of 5.6%, compared to a 16.7% of the one based on global features and a 21.8% for the HMM. Thus, we may conclude that the signature recognition technology based on DTW is not only more accurate (see Fig. 4) but also more robust to aging.
- The effect of aging may also be observed in the worsening of the scores variance through time, that is, the scores are not only lower but also more disperse. This way we can see how the variance increases around 45% from experiment A to E for all the three technologies tested.
- Another important observation to be made from the results shown in Fig. 6 is that the effect of aging on the signature trait is not negligible. There is a significant drift in the genuine score distributions (from the first to the last signature test set) in a relatively short period of time (15 months).

#### Objective 2: Aging user-dependency analysis

The sets of genuine scores generated in the previous experiments (Sect. 0) are used here to determine if certain users are more prone to suffer from aging. For this purpose we compute an Aging Coefficient (AC) defined as: 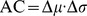, where 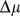 and 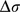 are respectively the mean and variance relative variation between two sets of scores. This way both aging effects (i.e., decrease of the genuine scores mean value and increase of the variance) are taken into account in one metric, so that the higher the AC of a user, the more affected that subject's signature is by the elapse of time.

**Figure 7:**
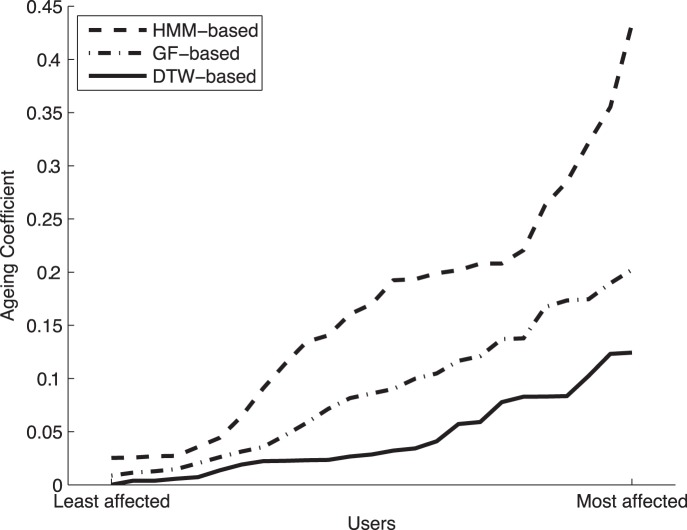
Aging Coefficient (AC) from the least affected to the most affected user by aging in the Signature Long-Term DB, for the three systems considered in the experiments. Please note that the least affected user, the most affected user, or any of the users in between, do not necessarily have to coincide (i.e., be the same signer) for all three systems. The three AC curves are shown on the same figure for an easier visual comparison across systems.

The AC is computed for all the users in the database between the genuine scores of experiments A and E, which are the two score distributions more separated in time. In Fig. 7 the AC is shown for all the subjects ordered according to their level of aging, from the lowest to the highest, for all the three systems considered in the experiments. Please note that the least affected user, the most affected user, or any of the users in between, do not necessarily have to coincide (i.e., be the same signer) for all three systems. The three AC curves are shown on the same figure for an easier visual comparison across systems.

The five most/least affected subjects by aging (i.e., those with respectively a higher/lower AC) are shown in Table 4 for all the three systems tested. For completion, the individual mean and variance variation indexes (i.e., 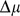 and 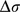) are also given.

**Table 4:** Most and least affected users by aging in the Signature Long-Term DB according to the three systems considered in the experiments.

		Aging: user dependency	
		Most affected users	Least affected users
	Δ*μ*	15, 17, 16, 22, 4	19, 27, 3, 9, 28
HMM	Δ*σ*	17, 4, 5, 26, 12	28, 6, 1, 9, 3
	AC	17, 4, 5, 22, **11**	28, **3**, 6, 27, **19**
	Δ*μ*	16, 24, 11, 23	19, 21, 3, 2, 27
GF	Δ*σ*	14, 1, 21, 6, 9	18, 12, 16, 17, 13
	AC	**1**, 24, **7**, **11**, 21	18, 12, 21, **19**, **3**
	Δ*μ*	7, 16, 11, 1, 8	19, 13, 3, 14, 26
DTW	Δ*σ*	11, 9, 16, 14, 2	19, 24, 26, 8, 29
	AC	16, **11**, **1**, 18, **7**	**19**, 13, 26, **3**, 24

**Figure 8:**
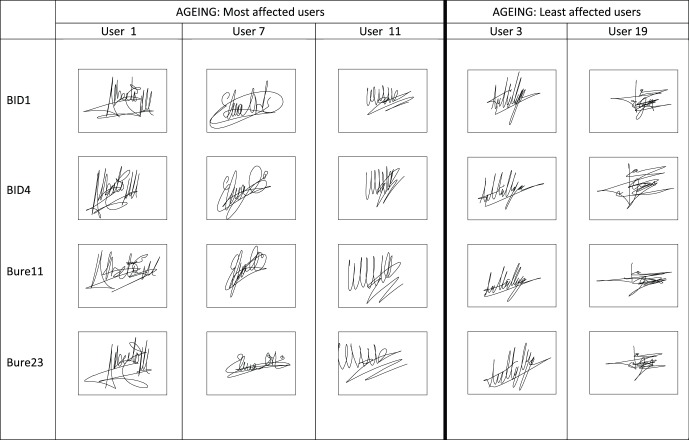
Most (left) and least (right) affected users by aging in the Signature Long-Term DB according to Table 4.

Users with the most appearances in the AC rows (in bold) are depicted in Fig. 8.

Different observations may be extracted from the results shown in Fig. 7 and Table 4:

- As expected, not all the systems present the same AC values. The DTW-based system has the lowest values (i.e., most consistent system over time), compared to the one based on global features (GF-based) and the HMM. This is consistent with the results obtained in Sect. 0 and confirms that the AC is a valid metric to evaluate the level of aging.
- In all the three systems there is a very big difference (around 95%) between the AC of the least and most affected users. Thus, even for the most robust technologies (DTW), the degree of aging is very dependent on the signer.
- In general the users tend to perform consistently well (3, 19) or badly (1, 17, 11) regardless of the recognition system used. Furthermore, none of the top five users in a system (i.e., those least affected by aging) appear in the list of the worst five users of the other two systems, and vice versa. This means that, as a general rule, a subject that despite of the aging effect presents high recognition rates on a given system, will be very likely to be consistently recognized if the system is changed.

Therefore, we may conclude that, although some technologies are more robust than others to aging, the degree of deterioration of a subject's signature depends mainly on the subject and not on the recognition system being used.

Those subjects with the highest number of appearances in the AC rows of Table 4 (shown in bold) are considered to be those with a more/less stable signature. The signatures of these users are depicted in Fig. 8 where we can see that the complexity of the signature is not a key factor in the level of aging. That is, complex signatures (i.e., long signatures, with the written name and flourish) may be very affected by aging or, on the contrary, can also be very stable through time. The same happens for short and simple signatures. In other words, these initial results seem to suggest that the degree of aging does not depend on the type of signature, but on the signer. However, these findings regarding aging and signature complexity should be further addressed on a specific database where signatures are classified into different complexity groups by expert examiners.

#### Objective 3: Template update analysis

The results presented in Sects. 0 and 0 confirm the necessity to develop strategies that can help to minimize the effect of aging, especially in those behavioral or learned traits, such as the signature, which are more sensitive to time. Here, we analyze the efficiency of different template update approaches varying the enrollment signatures used to compute the users models and testing always with the same set of samples, as shown in Table 5. In particular, the scenarios considered are:

**Table 5:** Enrollment and test signatures used to compute the genuine scores in the template update experiments.

	Template Update Experiments	
	Enrollment	Test
Exp. F (baseline)	BID1 (4 sig.)	Bure13
Exp. G (complete)	Bure11 (4 sig.)	Bure13
Exp. H (mixed)	BID1 (4 sig.)+Bure11 (4 sig.)	Bure13
Exp. I (complete)	Bure11 (4 sig.)+Bure12 (4 sig.)	Bure13

- Baseline result (Exp. F). This represents the scenario with no template update strategies to correct aging. There is a 14 month difference between the enrolled model (BID1) and the test set (Bure13).
- Complete update (Exp. G). The first template update approach considered is to discard the old enrollment samples (BID1) and replace them by new samples acquired very close in time to the test set (Bure11).
- Mixed update (Exp. H). In this case we do not discard the old samples but we update the enrolled model with newly acquired samples (BID1+Bure11). Thus, in this scenario there will be more available data to train the model than in the previous two cases (experiments F and G).
- Complete update (Exp. I). Here, we consider the same amount of training data as in experiment H, but all of it comes from recent acquisitions (Bure11+Bure12).

**Figure 9:**
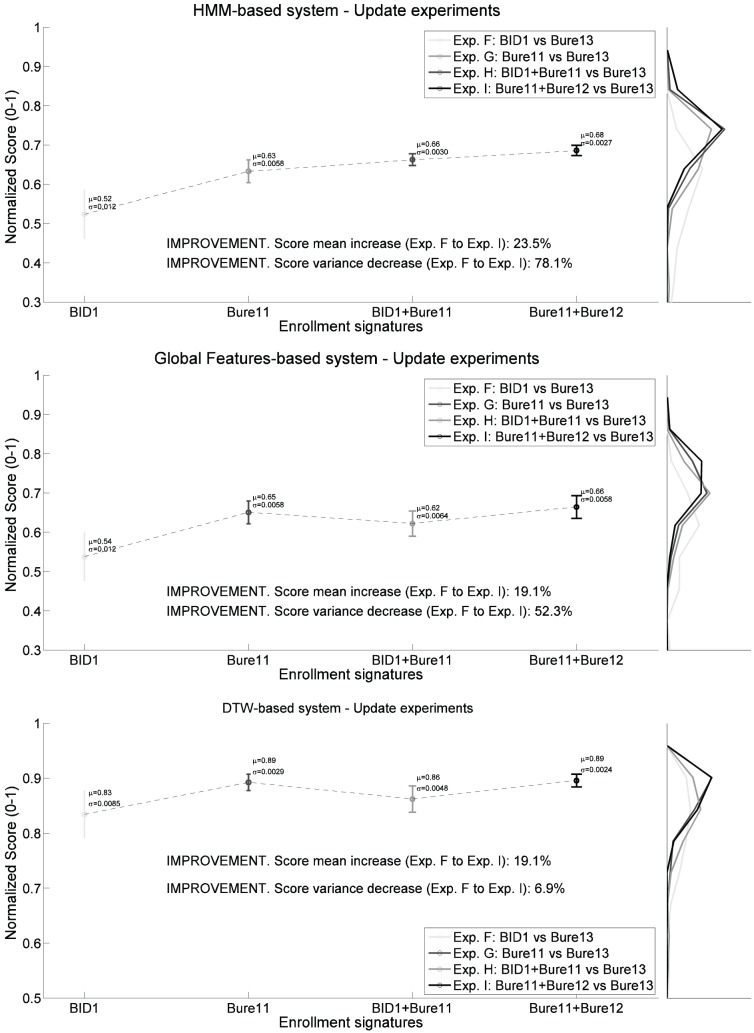
Mean (circles) and variance (vertical lines) of the genuine score distributions (in vertical on the right) for the 4 different template update strategies tested and for the three systems considered in the experiments. A darker gray shade represents a better performance of the given system.

The results of the previously described setups for the three considered systems are shown in Fig. 9. As in the case of the aging experiments the score distributions for each of the four considered scenarios is shown on the right planes in vertical with a darker gray shade representing a better performance of the given system. On the left plane we can see the evolution of the mean (circles) and variance (vertical lines) of the score distributions. Although all the template update strategies studied improve the performance with respect to the baseline experiment (in all cases there is an increase of the mean value and a decrease of the variance), two different behaviors may be observed in Fig. 9 depending on the signature recognition system considered:

- HMM system. HMM-based systems heavily depend on the amount of training data available [54]. As a consequence, it is better to perform a mixed update (i.e., do not discard the old samples, exp. H) so that the model is trained with as many signatures as possible (8 signatures, in this particular case), instead of using few recent samples (i.e., exp. G, where only 4 signatures are used for enrollment).
- Global features and DTW systems. On the other hand, the systems based on DTW and global features do not rely as much on the amount of enrollment data, but on the quality of these data [59]. Therefore, the performance reached using 4 recently acquired samples (exp. G) is almost the same as the one obtained using 8 of those signatures (exp. I). This means that, as can be seen in Fig. 9, in these cases it is preferable to perform a complete update with the most recent samples (i.e., exp. G) than to keep the old ones (i.e., exp. H) even if this means training the enrolled model with a smaller number of signatures.

As could be expected, in all cases the best possible template update strategy is to use for enrollment all the most recent samples available (i.e., exp. I). However, this may represent a somewhat unrealistic scenario, as we are assuming that we have access to as many as 8 signatures captured in a time period very close to the test set. The amount of new collected data will rarely comply with this condition.

### Tests 2: Signature Evolution

The results presented in Sect. 0 clearly show that the effect of aging is patent in the signature trait. The purpose of the present set of experiments is to further investigate the causes of the deterioration in the performance of signature recognition systems.

From a human perspective, the changes experienced with age by certain biometric traits are easily distinguished. For instance, we know that the face gradually loses its oval shape and that the wrinkles and sun-stains make its texture less smooth (in fact, these characteristics are successfully used for automatic age estimation purposes). However, what are the changes and transformations, if any, undergone by signatures with age?

In order to shed some light on this difficult question, the aging-related issues raised in this section are: How do signatures typically evolve over time? What type of transformations do they suffer? Are some signature-defining features more stable over time than others?

#### Objective 4: Signature evolution analysis

In order to determine the way in which signatures typically evolve with time, five of the most representative global features given in [56] (also in Appendix S1) have been analyzed for the whole Signature Long-Term DB. Not all the features proposed in [56] have a direct physical meaning, thus, the selected parameters have been those with an easy interpretation, namely: duration of the signatures (parameter 1 in [56]), number of maxima points in 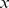 (parameter 8) and 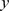 (parameter 12), number of pen-ups (parameter 2) and the average speed (parameter 26).

**Figure 10:**
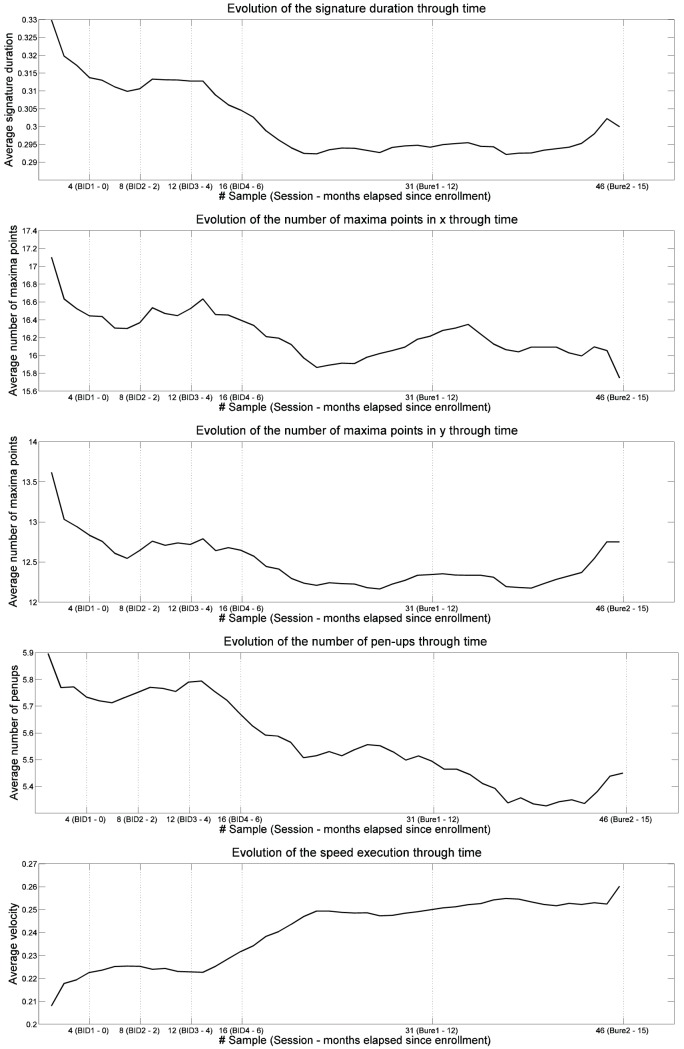
Evolution through time of the duration, maxima points in *x*, maxima points in *y*, number of penups and speed of the signatures in the Signature Long-Term Database.

These parameters have been averaged for all the users in the database in a sample by sample basis. That is, in the end, for each of the features, a 46-dimensional vector is computed where each element is the result of averaging the value of that parameter for the corresponding sample (from 1 to 46) of all the users in the database. In that way, we can see the evolution of the feature value from the first acquisition (month 0) to the last one (month 15). The results are shown in Fig. 10.

We can observe that, regardless of the user, the general trend for the signatures is to become: shorter, with fewer singular points and penups, and faster. That is, the results imply that signatures tend to be simplified with time.

#### Objective 5: Parameter evolution analysis

In this case the goal is to determine which of the global features proposed in [56] are more stable through time and, on the contrary, which are those that suffer the largest variations in the long term. For this purpose we use a Variation Coefficient (VC) analogue to the Aging Coefficient (AC) computed in Sect. This new Variation Coefficient is defined as: 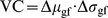, where 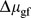 and 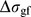 are respectively the mean and variance relative variation of a certain global feature between two acquisition sessions.

Prior to compute the VC, the values of the global features are averaged for all the users in the database on a sample by sample basis. That is, for each sample (1–46) we compute a 100-dimensional vector where each dimension is the mean value of that global feature for all the users in the dataset. Then, in order to evaluate the degree of variation through time of each global feature, the VC is computed between the samples of acquisition sessions BID1 and Bure2, which are the two most distant in time.

**Figure 11:**
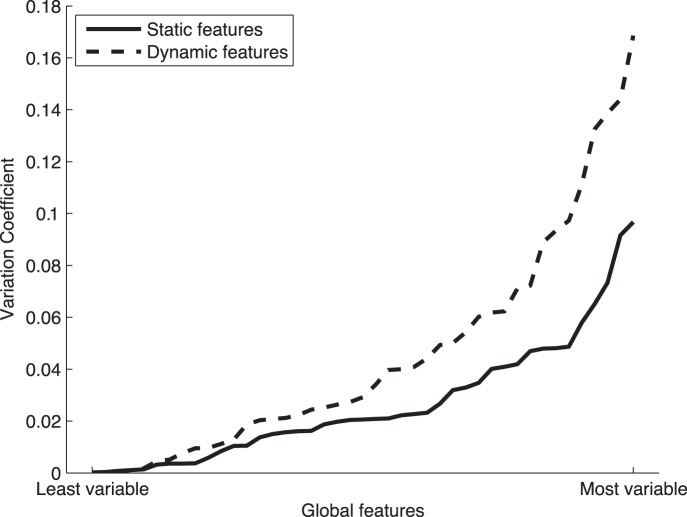
Variation Coefficient (VC) from the least variable to the most variable dynamic and static features (see Table 1) proposed in [56] (see Appendix S1).

In Fig. 11 we show the value of the Variation Coefficient from the least variable to the most variable static and dynamic features. On the other hand, in Table 6 the 10 most and least variable features are shown following the numbering criterion used in [56]. The ‘S’ and ‘D’ stand for Static and Dynamic features respectively, according to the classification given in Table 1.

**Table 6:** Most and least variable features over time.

	Most variable global features
Δ*μ_gf_*	33(D), 36(S), 47(D), 95(S), 66(S), 64(D), 31(D), 10(D), 76(D),85(D)
Δ*σ_ gf_*	73(S), 86(S), 76(D), 19(S), 85(D), 13(D), 90(D), 77(S), 65(S), 28(S)
VC	33(D), 47(D), 76(D), 85(D), 10(D), 64(D), 31(D), 36(S), 9(D), 32(D)
	**Least variable global features**
Δ*μ_gf_*	38(D), 59(D), 3(D), 17(S), 20(D), 7(S), 19(S), 40(D), 46(S), 60(D)
Δ*σ_ gf_*	93(S), 72(S), 58(D), 45(D), 17(S), 97(S), 21(D), 62(D), 67(S), 54(S)
VC	17(S), 58(D), 38(D), 93(S), 59(D), 72(S), 3(D), 45(D), 97(S), 7(S)

The numbering criterion is the same used in [56] (also in Appendix S1). ‘S’ stands for Static and ‘D’ for Dynamic according to the classification established in Table 1.

In Table 6 we can see that 9 out of the total 10 most unstable features correspond to parameters measuring dynamic information. Furthermore, Fig. 11 shows how, in general, dynamic features present a higher variability with time. From these results it may be concluded that the static information of a signature (e.g., geometric, spatial, or angular) is more robust over time than the dynamic data (e.g., velocity or acceleration). In other words, with time, signers tend to be more consistent repeating the shape of their signature rather than the way in which this shape is produced. These results are in line with the findings of previous related studies [6], [21], [61].

## Limitations of the Study, Open Questions, and Future Work

The main limitations of the present study are derived from the characteristics of the database used in the experiments. It has been mentioned in the article that the On-Line Signature Long-Term DB is, as far as the authors are concerned, unique regarding the number of subjects whose signature has been uniformly tracked over more than a year. Nevertheless, although this was the best available possibility, it is still limited both in terms of individuals (29) and time span considered (15 months).

The present work sets a first landmark in the understanding of aging in a behavioral biometric. However, its conclusions still have to be confirmed by further analysis and assessment on databases comprising a big number of uniformly-acquired samples for a larger number of individuals (several hundreds) and over a longer period of time (several years). However, we do believe that the experimental protocol and posterior analysis carried out in the present work is general and may serve as a baseline to be applied in future studies.

Therefore, the results, findings and conclusions presented in the article should be taken as a first approximation to the challenging problem of assessing aging in the signature trait, but not as conclusive and demonstrated facts. Furthermore, the study is also constrained to the type of subjects present in the database: Spaniards white Caucasians, mostly between 20 and 25 years of age, with a higher education degree (or pursuing it). For similar studies concerning other sectors of the population, specific data should be acquired.

Accordingly, the present study should be understood as a valuable but limited start which leaves different open questions which should be addressed in similar future works. For instance:

- Is 15 months a sufficiently long period of time to be in the presence of real “aging”? Although all the results given in the present work point in that direction, as mentioned above, this end should still be fully confirmed on a database acquired over a larger time span.
- What is the relationship (if any) between signature complexity and aging? In the current work an initial approach to address this issue has been established. However, more rigorous studies should be carried out on databases where signatures have been grouped into different complexity levels either by experts, different human observers, or some type of objective measure.
- Can the results presented here (using data acquired in laboratory conditions) be generalized to real world scenarios? For this type of study specific data from a real application should be employed.
- Are the signatures from men/women more prone to aging? A large gender-balanced database may be used to study this issue.
- Is the aging effect more pronounced in individuals with low writing skills? The current study was carried out only taking into account subjects with higher education degrees.

### Conclusions

We have conducted the first systematic study on the degradation of on-line signature with time and how this aging effect may be compensated. For this purpose, we have introduced the Signature Long-Term DB which contains the dynamic signature samples of the 29 common users of the BiosecurID and the Biosecure databases. All the subjects were captured under very similar conditions over a 15 month time span. The experiments, carried out using three totally different state-of-the-art systems representing the most usual technologies in on-line signature recognition, have proven that the aging effect is present in this trait even for time lapses of several months. Several conclusions have been extracted throughout the work thanks to the consistent and reproducible experimental protocol followed:

- Aging in the signature trait is a user-dependent effect. This means that:

- In general, a user affected by aging perform badly regardless of the system being used (this deterioration will be higher in those systems more sensitive to time).
- Complex and simple signatures can present the same amount of aging. Aging does not seem to depend on the type of signature but on the signer.

- Not all signature recognition technologies are equally affected by aging. The one based on DTW has demonstrated that it is not only the most accurate [60], but also the most robust to the passing of time.
- Global features containing dynamic information are in general less stable with time than those which comprise static information.
- With time, signatures evolve towards a higher simplicity. They become: shorter, faster and with fewer singular points and pen-ups.
- Depending on the signature recognition system being used some template update strategies are more efficient than others.

In summary, due to its very high user-dependency, the analysis and subsequent correction of aging in the signature trait should be done, ideally, on a user by user basis. Given a specific signature recognition technology, different template update approaches should be adopted for different users, depending on the performance degradation that each of the subjects present with time. This is consistent with previous research works which also emphasize the strong user dependencies found in signature recognition [62], [63].

In light of the experimental results obtained in the present work, a possible strategy to detect the appearance of aging in the signature of a given individual would be to follow a constant monitoring over time of the Aging Coefficient. A possible “aging detection” protocol for a signature-based application would be:

1. Set a suitable AC threshold (i.e., 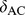) for the given application depending on the amount of aging allowed.
2. With every new genuine access attempt, estimate the mean and variance of the last known 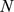 genuine access attempts and compare them to the mean and variance of the first 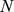 attempts (i.e., attempts that were recorded when the individual first started using the application).
3. Given the variation of the mean and variance between both sets of scores (new and old) compute the AC.
4. If 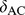 is exceeded, apply a suitable template update strategy depending on the signature recognition technology being used.

In this suggested protocol both 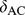 and 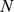 will depend on the type of application where it is being implemented (e.g., high security, commercial, high convenience), and on the level of restriction that will be imposed on aging. If only a small amount of aging is allowed a small value of both variables should be selected. On the contrary, if the designer prefers to be quite flexible with aging, larger values would be acceptable.

Research works such as the one presented here try to shed some light into the difficult problem of biometric aging. Performing systematic studies of biometric systems sensitivity to time is essential before effective strategies that minimize the impact of the detected effects can be developed, so that the user acceptability of this rapidly emerging technology is improved.

This way, we believe that this work can be of great utility not only for researchers, but also for developers and vendors in order to produce more secure and trustful applications based on the signature trait, to better understand its strengths, and to be able to foresee the weaknesses of this biometric modality. Furthermore, this type of study can also help to develop the ongoing biometric standards and to better define the requirements that real applications should comply with [64]–[66].

In summary, the work main contribution is the theoretical and practical new knowledge built in the fields of signature recognition and biometric aging, which may be directly applied by researchers and companies for the future development of the biometric technology.

## Supporting Information

Appendix S1 (ZIP)
