# Supplementary material for: Aging in Biometrics: An Experimental Analysis on On-Line Signature
Source: PLoS One. 2013 Jul 23;8(7):e69897. doi: 10.1371/journal.pone.0069897 (PMC3720939; doi:10.1371/journal.pone.0069897)
Supplement: Appendix S1 — (ZIP) [file pone.0069897.s001.zip › SupportingInformationFile_AppendixS1.pdf]

Aging in Biometrics: An Experimental Analysis on On-Line Signature

Javier Galbally[1,*], Marcos Martinez-Diaz[1], Julian Fierrez[1]
1 Biometric Recognition Group-ATVS, Universidad Autonoma de Madrid. Madrid. Spain.
∗ E-mail: javier.galbally@uam.es

Appendix S1. Set of Global Features

Since it is largely referenced throughout the present article, Table 1 contains the whole set of 100 global features which was first proposed in [1] as a parametrization to model on-line signatures. This way the reader may have quick reference to the parameters. For further details on the features please refer to the work mentioned above [1].

References

1. Fierrez-Aguilar J, Nanni L, Lopez-Pealba J, Ortega-Garcia J, Maltoni D (2005) An on-line signature verification system based on fusion of local and global information. In: Proc. IAPR Audio- and Video-Based Biometric Person Authentication (AVBPA). Springer LNCS-3546, pp. 523-532.

Table 1: Set of global features proposed in [1] sorted following the same order used in that work (T denotes time interval, t denotes time instant, N denotes number of events, and θ denotes angle). Note that all notations are either defined or referenced somewhere in the table (e.g., j is defined and referenced in **4**, Δ is defined in 15, histograms in $51, 61, 70, 93, \ldots$ are referenced in 34, etc.).

Ranking	Feature Description	Ranking	Feature Description
1	signature total duration T_s	2	N(pen-ups)
3	N(sign changes of dx/dt and dy/dt)	4	average jerk $\bar{j}$
5	standard deviation of a_y	6	standard deviation of v_y
7	(standard deviation of y)$/\Delta_y$	8	N(local maxima in x)
9	standard deviation of a_x	10	standard deviation of v_x
11	j_{rms}	12	N(local maxima in y)
13	t(2nd pen-down)$/T_s$	14	(average velocity $\bar{v}$)$/v_{x,\max}$
15	$\frac{A_{\min}=(y_{\max}-y_{\min})(x_{\max}-x_{\min})}{(\Delta_x=\sum_{i=1}^{\text{pen-downs}}(x_{\max\,\lvert i}-x_{\min\,\lvert i}))\Delta_y}$	16	$(x_{\text{last pen-up}} - x_{\max})/\Delta_x$
17	$(x_{\text{1st pen-down}} - x_{\min})/\Delta_x$	18	$(y_{\text{last pen-up}} - y_{\min})/\Delta_y$
19	$(y_{\text{1st pen-down}} - y_{\min})/\Delta_y$	20	$(T_w\bar{v})/(y_{\max} - y_{\min})$
21	$(T_w\bar{v})/(x_{\max} - x_{\min})$	22	(pen-down duration T_w)$/T_s$
23	$\bar{v}/v_{y,\max}$	24	$(y_{\text{last pen-up}} - y_{\max})/\Delta_y$
25	$\frac{T((dy/dt)/(dx/dt)>0)}{T((dy/dt)/(dx/dt)<0)}$	26	$\bar{v}/v_{\max}$
27	$(y_{\text{1st pen-down}} - y_{\max})/\Delta_y$	28	$(x_{\text{last pen-up}} - x_{\min})/\Delta_x$
29	(velocity rms v)$/v_{\max}$	30	$\frac{(x_{\max}-x_{\min})\Delta_y}{(y_{\max}-y_{\min})\Delta_x}$
31	(velocity correlation $v_{x,y}$)$/v_{\max}^2$	32	$T(v_y > 0 \mid \text{pen-up})/T_w$
33	$N(v_x = 0)$	34	direction histogram s_1
35	$(y_{\text{2nd local max}} - y_{\text{1st pen-down}})/\Delta_y$	36	$(x_{\max} - x_{\min})/x_{\text{acquisition range}}$
37	$(x_{\text{1st pen-down}} - x_{\max})/\Delta_x$	38	$T(\text{curvature} > \text{Threshold}_{\text{curv}})/T_w$
39	(integrated abs. centr. acc. a_{Ic})$/a_{\max}$	40	$T(v_x > 0)/T_w$
41	$T(v_x < 0 \mid \text{pen-up})/T_w$	42	$T(v_x > 0 \mid \text{pen-up})/T_w$
43	$(x_{\text{3rd local max}} - x_{\text{1st pen-down}})/\Delta_x$	44	$N(v_y = 0)$
45	(acceleration rms a)$/a_{\max}$	46	(standard deviation of x)$/\Delta_x$
47	$\frac{T((dx/dt)(dy/dt)>0)}{T((dx/dt)(dy/dt)<0)}$	48	(tangential acceleration rms a_t)$/a_{\max}$
49	$(x_{\text{2nd local max}} - x_{\text{1st pen-down}})/\Delta_x$	50	$T(v_y < 0 \mid \text{pen-up})/T_w$
51	direction histogram s_2	52	t(3rd pen-down)$/T_s$
53	(max distance between points)$/A_{\min}$	54	$(y_{\text{3rd local max}} - y_{\text{1st pen-down}})/\Delta_y$
55	$(\bar{x} - x_{\min})/\bar{x}$	56	direction histogram s_5
57	direction histogram s_3	58	$T(v_x < 0)/T_w$
59	$T(v_y > 0)/T_w$	60	$T(v_y < 0)/T_w$
61	direction histogram s_8	62	(1st $t(v_{x,\min})$)$/T_w$
63	direction histogram s_6	64	T(1st pen-up)$/T_w$
65	spatial histogram t_4	66	direction histogram s_4
67	$(y_{\max} - y_{\min})/y_{\text{acquisition range}}$	68	(1st $t(v_{x,\max})$)$/T_w$
69	(centripetal acceleration rms a_c)$/a_{\max}$	70	spatial histogram t_1
71	θ(1st to 2nd pen-down)	72	θ(1st pen-down to 2nd pen-up)
73	direction histogram s_7	74	$t(j_{x,\max})/T_w$
75	spatial histogram t_2	76	$j_{x,\max}$
77	θ(1st pen-down to last pen-up)	78	θ(1st pen-down to 1st pen-up)
79	(1st $t(x_{\max})$)$/T_w$	80	$\bar{j}_x$
81	T(2nd pen-up)$/T_w$	82	(1st $t(v_{\max})$)$/T_w$
83	$j_{y,\max}$	84	θ(2nd pen-down to 2nd pen-up)
85	$j_{\max}$	86	spatial histogram t_3
87	(1st $t(v_{y,\min})$)$/T_w$	88	(2nd $t(x_{\max})$)$/T_w$
89	(3rd $t(x_{\max})$)$/T_w$	90	(1st $t(v_{y,\max})$)$/T_w$
91	$t(j_{\max})/T_w$	92	$t(j_{y,\max})/T_w$
93	direction change histogram c_2	94	(3rd $t(y_{\max})$)$/T_w$
95	direction change histogram c_4	96	$\bar{j}_y$
97	direction change histogram c_3	98	θ(initial direction)
99	θ(before last pen-up)	100	(2nd $t(y_{\max})$)$/T_w$
